# Food-Based Nutrition Care Provided to Older Community-Dwelling Adults with or at Risk of Malnutrition Following Hospital Discharge—A Scoping Review

**DOI:** 10.3390/nu18172846

**Published:** 2026-08-31

**Authors:** Alita Rushton, Shelley Roberts, Jana Waldmann, Trent Payne, Norman R. Morris, Jack J. Bell

**Affiliations:** 1 School of Allied Health, Sport and Social Work, Griffith University, Gold Coast, QLD 4222, Australia; s.roberts@griffith.edu.au (S.R.); trent.payne@uq.edu.au (T.P.); n.morris@griffith.edu.au (N.R.M.); jack.bell@health.qld.gov.au (J.J.B.); 2 Allied Health Research, Gold Coast Hospital and Health Service, Gold Coast, QLD 4215, Australia; 3 Library Services, The Prince Charles Hospital, Chermside, QLD 4032, Australia; jana.waldmann@health.qld.gov.au; 4 Faculty of Health, Medicine, and Behavioural Sciences, The University of Queensland, Herston, QLD 4006, Australia; 5 Australian Frailty Network, Frazer Institute, Faculty of Health, Medicine, and Behavioural Sciences, The University of Queensland, Dutton Park, QLD 4102, Australia; 6 Allied Health Research Collaborative, The Prince Charles Hospital, Chermside, QLD 4032, Australia

**Keywords:** aged, aging in place, community-dwelling, continuity of patient care, food, food-based nutrition, malnutrition, nutritional status, nutrition therapy, post-discharge

## Abstract

**Background**: Malnutrition causes complications and adverse effects in older people and is a burden on healthcare systems. Older people with, or at risk of malnutrition who receive nutrition care in hospitals do not appear to be adequately engaged with post-discharge nutrition care processes. Food-based approaches (exclusive of oral nutritional supplements) are commonly considered the ‘first’ or frontline approach by dietetic professionals in the community setting. The aim of this scoping review is therefore to identify and synthesise the literature on food-based nutrition care provided to older community-dwelling adults who have been identified as malnourished or at risk of malnutrition during a recent hospital admission. **Methods**: This scoping review follows the methodological framework by Arskey and O’Malley, revised by the Joanna Briggs Institute. Results are reported according to the PRISMA-ScR guideline. Studies evaluating food-based nutrition care interventions provided to patients post-discharge from a hospital admission returning to a community setting were included in this review. **Results**: A total of 4086 records were screened, and eleven articles from seven independent studies were included. Highly heterogeneous findings may suggest improvements in weight, dietary intake, quality of life, physical function, anxiety and/or depressive symptoms, and reduction in readmissions associated with interventions including food-based nutrition care. However, sample sizes were generally small and evaluated as part of multicomponent care. **Conclusions**: Additional research is required to support frontline, food-based approaches for older community-dwelling adults, with or at risk of malnutrition, who are recently discharged from hospital. In the interim, multi-component nutrition interventions may be considered to improve nutritional intake.

## 1. Introduction

Malnutrition is a disease caused by inadequate protein and energy intake to meet individual needs. It leads to weight loss, muscle wasting, decreased fat stores, and reduced physical and functional status [1,2,3]. Malnutrition is prevalent in 20–50% of hospital inpatients [4]. Hospitalised individuals with malnutrition are more likely to develop complications and spend more time in hospitals and other healthcare settings, leading to increased morbidity and earlier mortality [4,5,6,7,8]. Health services are also burdened by malnutrition through prolonged length of stay, more frequent hospital readmissions, and increased health service utilisation, contributing to substantially increased healthcare costs [6,8,9,10].

To date, nutrition care processes, improvement efforts, and research for people with malnutrition have remained largely centred around acute hospital settings and the inpatient nutrition care process [7,11,12]. However, many older people admitted to hospital are already malnourished on admission [1]. Short hospital lengths of stay, combined with the complexity of medical conditions, treatment side-effects, interruptions to nutrition care, and procedure-related fasting means that nutritional status improvements are unlikely to be achieved during inpatient care alone [13]. Hospitalised patients with acute illness often experience both inadequate intake and reduced mobility; these have been long recognised for accelerating skeletal muscle loss resulting in sarcopenia [14,15,16]. Older adults are especially vulnerable to hospital-induced sarcopenia with slow recovery of muscle mass and function [17,18]. Consequently, explicit recommendations are provided in key recommendations, guidelines, and models of care for continuation of nutrition care after discharge from hospital [7,19,20].

In community-dwelling older adults, malnutrition prevalence ranges from 3 to 20% depending on the diagnostic tool used and setting studied [21,22,23,24,25,26]. The primary determinants of malnutrition in older people include inadequate intake, increased requirements, and reduced nutrient bioavailability [27]. These determinants frequently co-exist. For example, chronic illness combined with anorexia of ageing is commonly observed in older adults and the effects are often mutually reinforcing [6,28,29]. Many other commonly observed risk factors for malnutrition appear more relevant to usual place of residence than acute hospital admissions, such as food insecurity, decreased food availability or access, socioeconomic, educational, and psychological factors, and food-related behaviours [5,6,30,31,32,33,34].

Although data are limited, clinicians, systems, and older people with or at risk of malnutrition who receive nutrition care in hospitals do not appear to be adequately engaged with post-discharge nutrition care processes [35,36,37]. A recent study found that under half of adult inpatients with or at risk of malnutrition across nine Australian hospitals reported having an ongoing plan for nutrition care or nutrition follow-up, and those that did were typically younger patients [38]. More broadly, across diverse healthcare systems, continuity of nutrition care after hospital discharge is often fragmented, and requires consideration of context-specific barriers and enablers to bridge the gap between hospital and community settings [39,40,41,42].

This gap may be partly attributable to limited research and the lack of evidence-based recommendations specifically addressing nutrition care for community-dwelling adults following hospitalisation [6,43]. The strongest evidence available is for Oral Nutritional Supplements (ONS) and parenteral nutrition. Consequently, recommendations for nutrition after discharge from hospital appear limited to provision of ONS and enteral/parenteral nutrition support [7,11]. This approach may be problematic in practice, with recent evidence demonstrating poor adherence to ONS strategies following discharge. A recent single-site study reported that only one-third of patients diagnosed with malnutrition accepted ONS post-discharge from hospital [35]. Other studies have identified barriers to access and adherence to ONS in the community setting, including cost and limited patient awareness of the importance of nutrition [26,36].

As an alternative approach, a recent scoping review examining the effectiveness of individualised nutritional care plans developed during hospital admissions with follow-up for three months post-discharge reported mixed findings [44]. Four of the nine included studies demonstrated a significant effect of the intervention on nutritional status while another four of the studies showed no effect [44]. The interventions predominately consisted of dietary plans, advice, and counselling, and did not include food-based nutrition care interventions such as ready-made meals.

Food-based approaches are commonly considered the ‘first’ or frontline approach by dietetic professionals in the community setting [7,26,45,46]. However, to date there is no synthesised literature (e.g., scoping or systematic reviews, evidence-based practice, care guidelines) considering the effects of food-based nutrition interventions for older adults with or at risk of malnutrition following discharge from hospital, and evidence appears limited to a small number of studies [47,48,49].

The aim of this scoping review is therefore to identify and synthesise the literature on food-based nutrition care provided to older community-dwelling adults who have been identified as malnourished or at risk of malnutrition during a recent hospital admission.

## 2. Materials and Methods

This scoping review was undertaken following the Joanna Briggs Institute (JBI) reviewers manual [50] founded on the methodological framework by Arskey and O’Malley [51] revised by JBI. Results are reported according to the PRISMA-ScR reporting guideline [52]. Methodological principles of the Two-Week Scoping Review guidelines were followed; however the review was not completed within the recommended two-week timeframe [53]. This scoping review was registered with OSF on 16 April 2024, including associated protocol which can be found at https://doi.org/10.17605/OSF.IO/F65DR.

### 2.1. Eligibility Criteria

#### 2.1.1. Participants

Manuscripts from primary research studies were included if participants (or the majority of participants) were older persons recently discharged home from hospital and residing in the community (including independent living units at a retirement village) and identified as malnourished or at risk of malnutrition. Current hospital inpatients and those living at residential aged care facilities or long-term care were excluded. Gender, geographic location, and sociocultural status were not limited.

#### 2.1.2. Concept

All primary studies investigating food-based nutrition care were included. For the purposes of this review, we have defined ‘food-based nutrition care’ as the provision of food-based interventions in procuring, purchasing, preparing, and/or providing foods, fluids, or meals. This working definition was inclusive of meal purchasing and delivery, food fortification (such as supermarket-purchased foods or fluids that have additional protein or calories that do not meet the below definition of ONS), and provision of foods and/or fluids to people’s homes (e.g., partially prepared, fully prepared, or frozen meal delivery services). Our definition of food-based interventions excluded studies that only considered ONS, defined by the European Society for Clinical Nutrition and Metabolism (ESPEN) guidelines as ready-to drink liquids, cremes, or powder supplements that can be prepared as drinks or added to drinks and foods [11].

Multicomponent intervention studies that included food-based nutrition care and one or more additional nutrition care approaches (e.g., meal planning, support at mealtimes, education and counselling, ONS, enteral/parenteral nutrition, as well as food-based nutrition) were also included. Multicomponent interventions that did not include an element of food-based nutrition care were excluded (i.e., ONS or enteral/parental nutrition exclusively, or interventions combining ONS and dietary counselling without food-based nutrition care as defined above).

#### 2.1.3. Context

Studies evaluating food-based nutrition care interventions provided to patients post-discharge from a hospital admission, in a community setting, from any country were included. Studies solely in hospital or other inpatient healthcare settings were excluded. Only peer-reviewed studies were included with no limitation on study design or sample size. Review protocols, conference abstracts, theses, and non-peer-reviewed publications were excluded. Clinical trial protocols and reviews were included for reference list searching only.

### 2.2. Information Sources and Search Strategy

The search strategy was developed by the lead author (AR) and health librarian (JW), in consultation with field experts (SR, J.J.B). All authors confirmed the search strategy, and refinements were made as required. The search strategy was translated for each database by JW. Databases searched included MEDLINE (Ovid), Embase (Elsevier, Embase.com), APA PsycInfo (EBSCOhost), CINAHL (EBSCOhost), and Cochrane Central Register of Controlled Trials (CENTRAL) (Wiley). Other trial registries were not searched. The database searches were initially completed on 5 April 2024, and then re-run on 1 September 2025. SpiderCite (https://sr-accelerator.com/#/spidercite (accessed on 19 April 2024).) was used to identify additional articles by backward and forward searching each study included at the full-text stage. The bibliographies of any relevant systematic reviews were hand searched for additional studies. The search strategies for all databases can be found in Supplementary File S1. Google Advanced was used to identify grey literature (e.g., government reports) by searching for a predefined search string and limiting to a planned selection of government/organisation websites and publication types (PDF and Word documents). Grey literature search terms can be found in Supplementary File S1. Subject experts were not contacted for additional studies or data. No restrictions were placed on language or year. For records not published in English, DeepL Translator was used for title/abstract screening. Results were imported into EndNote 21 and de-duplicated using EndNote’s inbuilt duplicate identifier.

### 2.3. Study Selection/Screening

Title/abstract and full-text screening was undertaken independently by two authors (AR, TP) according to eligibility criteria. Papers without an abstract were screened based on title only. The first 100 results were pilot screened together by both authors (AR, TP). Following title and abstract screening, full texts were retrieved for the articles identified to proceed to full-text screening. Disputes were resolved by consensus and input from a third author (SR) when required. Title and abstract screening processes were undertaken using Screenatron, full-text screening processes were undertaken using EndNote, and Disputatron was used for title and abstract and full-text dispute resolution [54].

### 2.4. Data Charting

A data extraction table was developed by AR using Microsoft Excel^®^. It was pilot tested by AR and SR on one study with no modifications. Following registration, data extracted were author, year, country, study aim, study population and sample size, methodology/study design, intervention, outcomes assessed, and results. Data charting was undertaken independently by a single reviewer (AR) with each entry reviewed by SR or J.J.B. Minor adjustments were made, and any major adjustments required were discussed with the authorship team and the extraction table updated accordingly. Inter-reviewer agreement was not assessed.

## 3. Results

### 3.1. Search Results

The initial search was completed 1 April 2024. Screening commenced on 8 April whilst awaiting protocol registration with the aim of meeting the 2-week scoping review deadline. Registration was not obtained until 16 April. Data charting was delayed until registration. Delay to formal registration, combined with the requirement for a PhD student to take ownership of the majority of work, resulted in the rapid review timeline being abandoned. Initial forward and backwards screening disputes were finalised 23 May, with full team approval of initial papers for inclusion achieved on 3 June 2024. A final search re-run on 1 September 2025 yielding one additional study for inclusion.

**Figure 1:**
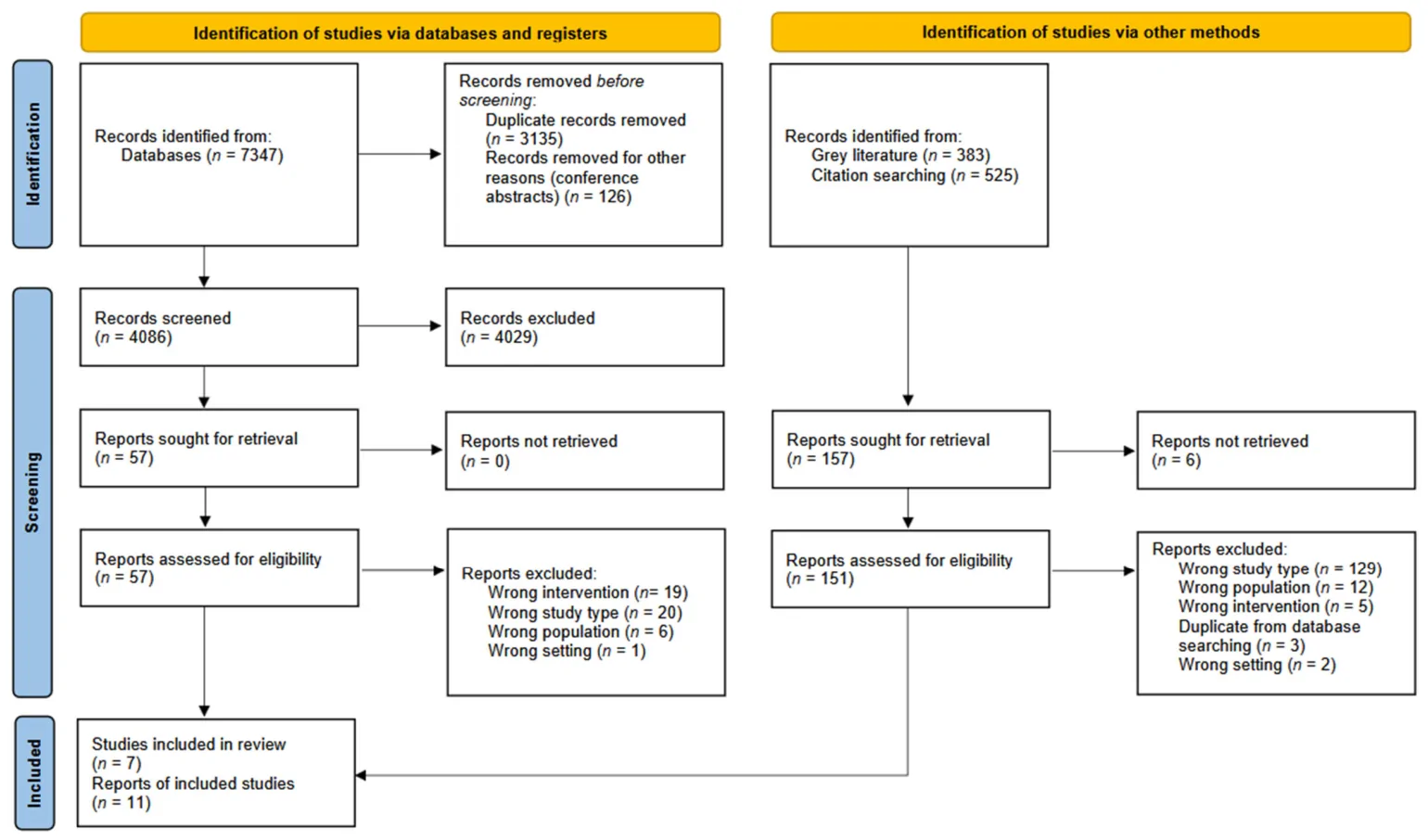
PRISMA flowchart.

In total, 7347 records were identified through database searches; 3135 duplicates and 126 conference abstracts were removed, 4086 records were screened, and eleven publications from seven studies were included (one study had four associated manuscripts, and another study had two associated manuscripts); of the 525 records searched via citation searching and 383 records searched via grey literature, no additional studies were included. One systematic review found through citation searching was identified as relevant to the topic studied. Hand searching of included studies of that review did not yield any further primary research studies for inclusion. The associated PRISMA flowchart can be found in Figure 1 [55].

### 3.2. Study Characteristics

A summary of included studies can be found in Supplementary File S2. Of the seven included studies, three were undertaken in the United States of America (USA), one in Iceland, two in Denmark, and one in Israel. Four studies (eight manuscripts) were randomised controlled trials (RCTs) [47,49,56,57,58,59,60], one was a mixed methods quasi-experimental, non-randomised controlled feasibility trial with embedded qualitative interviews [61], one was a pilot RCT [62], and one was a random order crossover trial [63]. Of the four RCTs, three compared the intervention to ‘usual care’, one provided an intervention for those in the control group in the form of an educational booklet, and the pilot RCT compared two different intervention groups (one receiving only ONS, one receiving only snacks). The mixed methods quasi-experimental, non-randomised controlled feasibility trial tested technology-supported home-delivered main and mid-meals (ordering meals and snacks through an electronic tablet) against usual care. The random order crossover trial compared provision of 7 medically tailored meals per week with 21 medically tailored meals per week.

All studies included investigated the impact of the intervention on the specified patient outcomes. Outcomes frequently assessed included nutrition status, hospital readmission, mortality, weight/height/body mass index, dietary intake, quality of life, functional status, physical performance, depressive symptoms, and/or patient satisfaction. Three studies also considered feasibility and acceptability of interventions. Most follow-up periods were relatively short (Supplementary File S2).

### 3.3. Intervention Characteristics

Interventions varied across studies, with all interventions including some level of food provision following discharge from hospital. Additionally, some interventions also included other elements of nutrition care such as education, a tablet app to support meal preferences and ordering, individualised nutrition plans, a volunteer lunch companion, and dietitian home visits. The interventions provided in each study have been summarised in Supplementary File S2.

Intervention duration varied from the first day post-discharge up to 12 months following discharge. Results were unclear as to whether interventions with a longer duration found more significant results. However, one study compared receiving 7 meals per week with receiving 21 meals per week and found that the number of meals provided per week was not a significant predictor of changes in patient outcomes [63].

### 3.4. Summary of Findings

Included studies assessed the effects of their intervention on various patient outcomes, with results consistently suggesting improvements in weight, dietary intake, quality of life, physical function, anxiety and/or depressive symptoms, and reduction in readmissions for those receiving the intervention (Table 1 and Supplementary File S2). However, results were not always statistically significant or powered. The majority of studies were small, single-site studies with no reference to statistical power or reported as not adequately powered/considered subject to type 2 error.

For those studies that had statistically powered results, the findings showed increased energy and protein intake and lower weight loss in the intervention group compared to the control group [47,49,56]; improved body weight, Body Mass Index (BMI), lean body mass, mid-arm circumference, health-related quality of life Visual Analogue Scale (VAS) score in the intervention group [49,56]; significant difference of VAS score and depressive symptoms between intervention and control group at endpoint [49]; improved physical function and nutrition status score in the intervention group compared to control group [47,56]; reduced number of hospital readmission at 1, 6, and 12 months post-discharge compared to control group, and lower proportion of participants readmitted to hospital 1 and 6 months post-discharge compared to control group [48]; reduced length of stay of readmission at 1, 6, 12, and 18 months post-discharge compared to control group [48]; and improved sarcopenia risk at 1 and 2 months post-discharge, diet adherence for heart failure, and malnourished patients [63]. Of the studies that measured mortality only one found improvements, with survival at six months improving significantly in the intervention group compared to the control group [60].

The studies that assessed feasibility reported challenges undertaking these types of interventions among older adults with or at risk of malnutrition, who are returning home from a recent hospital admission [57,61,64]. One study took 12 months to recruit 24 patients to the study due to the restrictive (but seemingly appropriate) inclusion criteria [57]. Another study found recruitment difficult, had issues with retention, and found patient outcome data collection, particularly those related to physical function, burdensome for participants [64]. Lindhardt et al. reported concerns about recruitment bias, as the intervention was only found to be acceptable for people who lived on their own or cooked for themselves [61]. Disease severity was also mentioned to impact results regarding patient outcomes (e.g., hospital readmissions) and stratified randomisation was recommended for future studies [57]. The most common reasons participants withdrew or did not attend follow-up visits were weakness, burden of study (time and effort), disliking the food in the intervention, interruption of intervention on daily activities, hospital re-admissions during intervention timeframe, and death.

## 4. Discussion

This scoping review has synthesised the available literature on food-based nutrition care provided to older community-dwelling adults who were identified as malnourished or at risk of malnutrition and recently returned home from hospital. Results indicate that food-based interventions following hospital admission may improve weight, nutrition status, dietary intake, quality of life, physical function, anxiety and/or depressive symptoms, and reduce hospital admissions and mortality. However, most inclusions were small single-site studies, many are likely underpowered, and only seven independent studies (presented across 11 publications) were included from only four countries. The overall depth and scope of empirical studies were considered insufficient to provide definitive recommendations to clinical practice.

Despite this lack of strong evidence, international guidelines and societal positions, for example those from ESPEN and the Academy of Nutrition and Dietetics, support food-based approaches across the continuum of care together with other interventions as part of individualised, multidisciplinary, multicomponent nutrition care for people with or at risk of malnutrition [7,20,65,66,67,68,69,70,71,72,73]. The Academy of Nutrition and Dietetics’ 2024 Evidence-Based Nutrition Practice Guideline for older adults in long-term care and community settings also supports inclusion of enhanced meal provision, food fortification, accessible meal services, and community nutrition programs as food-based approaches [66]. This guideline also encourages consideration of ONS, dietitian-led care, and other interventions to be combined as multicomponent individualised interventions to increase protein and energy intake and improve nutrition status.

A systematic review of 80 studies associated with home-delivered meals for those aged 45 and over, not specific to persons with or at risk of malnutrition or post-hospital discharge period, generally supported home-delivered meal programs to improve dietary and nutrient intake, food security, and a positive influence on nutrition [risk] measures [74]. However, this study concluded that the impact of these programs on health-related outcomes, beyond nutritional outcomes based on self-reported measures, was difficult to ascertain. The results of our scoping review would appear to support these findings for older community-dwelling adults who were at risk of malnutrition and recently returned home from hospital, albeit in a smaller number of underpowered studies.

Other reviews also suggest benefits of home-delivered meal programs in supporting recovery from a hospital admission and associated health event [75]. Older adults recently discharged from hospital who were not participating in meal delivery programs had poorer outcomes relating to health status, activities of daily living, and depressive symptoms, and had more difficulties with shopping and cooking compared to those who were participating in such programs. However, these findings were not specific to people with or at risk of malnutrition [75].

Alternatively, though limited to hospitalised adults with or a risk of malnutrition, another and more recent Cochrane review considered the effectiveness of different nutritional interventions using an individual participant data network meta-analysis of RCTs [76]. The complex methodology aimed to compare multiple oral nutritional support strategies, including food-based modification/enrichment, dietary education and counselling, mealtime support, assistance and changes to food environments, ONS, and multicomponent interventions. This study suggests that ONS may reduce mortality and serious adverse events. Moreover, it was concluded that food-based approaches did not demonstrate significant or high-certainty effects on mortality and serious adverse events or other outcomes, and acknowledged the lack of certainty regarding the benefits and risks of different types of nutritional support [76]. Notably, we would argue that the limited number of studies we have identified in this scoping review does not provide the depth of literature required to apply a meta-analyses or other synthesised approach to articulate which of any multi-component strategies are likely to be effective for older adults returning home from hospital.

Clearly more research is required for older adults discharged from hospital with or at risk of malnutrition, to adequately examine effectiveness of food-based approaches in addition to, or in comparison with, other nutrition support strategies. No studies identified in our scoping review demonstrated harm or contradicted food-based approaches. Multicomponent interventions included in our scoping review suggest that food provision and importantly, additional nutrition support strategies (e.g., education, volunteer companion at lunch time, nutrition therapy via phone call or home visit, individual care plans or ONS) were more likely to demonstrate statistically significant improvements on participants’ physical, nutritional, and quality of life outcomes [47,49,56,57,60].

More broadly, food-based interventions, particularly where combined with other nutrition interventions such as education, companionship, education, community support or follow-up, or ONS appear to (i) be feasible [47,60,77]; (ii) positively influence patient-centred outcomes such as nutritional intake, assessment and body composition measures, complications, symptom control, social, emotional, physical and functional status, and quality of life [1,26,47,48,49,56,76,78,79,80,81,82,83,84,85,86,87,88,89,90,91]; and (iii) at least some of the time, demonstrate association with healthcare outcomes including hospital readmission, length of stay, time spent out of hospital or healthcare, and mortality [47,60,89].

Given the above, a pragmatic evaluation suggests that, based on existing literature, food-based interventions alone may be beneficial for patients with or at risk of malnutrition discharged from hospital, especially when combined as part of a multicomponent nutrition intervention. The key limitation of this study is that findings are restricted to only 11 publications from seven independent studies in four countries. These have precluded the ability to consider potential differences in healthcare systems, policy and funding models, access to food assistance programs or home-delivered meal services, socio-cultural factors, or consider generalisability to low- and middle-income countries. Sample sizes were generally small with the exception of the HOMEFOOD study (four separate papers included), and follow-up periods were relatively short (up to 6 months). Interventions and outcome measures were heterogeneous; the majority of studies included food-based interventions in combination with ONS, nutrition education, and other therapies. This precluded the ability to attribute outcomes to the food-based component. Finally, the certainty of the evidence was not evaluated using formal quality appraisal due to the planned rapid review process.

Consequently, existing research appears insufficient to draw strong conclusions or establish recommendations for practice regarding food-based nutrition interventions for older community-dwelling adults, with or at risk of malnutrition, following discharge from hospital. Studies included in this review that measured feasibility highlighted that recruitment, retention, and collection of outcome measure in this population were challenging. Noting this caveat, future research priorities should consider high-quality designs that consider food-based interventions either alone or together as part of multicomponent nutrition interventions. Given that nutrition-related outcomes alone are unlikely to justify guideline recommendations or implementation decisions, these should ideally evaluate clinically meaningful and health-economic outcomes as primary endpoints. Given that intervention burden appeared to be a major reason for withdrawal, future studies should also aim to minimise participant burden. Large-scale implementation studies or registry-supported implementation–effectiveness hybrid studies may provide pragmatic opportunities to evaluate both clinical effectiveness and feasibility [92].

## 5. Conclusions

Food-based approaches are commonly considered the front-line approach to nutritional support for older community-dwelling adults, with or at risk of malnutrition, who are recently discharged from hospital. This scoping review demonstrates that the current evidence base for food-first approaches remains limited and heterogenous. Our only recommendation we can make is that well-designed intervention studies are required to justify food-based approaches. In the interim, teams and professional organisations should consider whether food-based approaches should continue to be considered front-line or ‘first’ approaches versus being included as part of multidisciplinary, multicomponent nutrition interventions to improve nutritional intake.

## Figures and Tables

**Table 1:** Summary of results.

Study	Design	*n*=	Intervention Type and Duration	Dietary Intake	Nutritional Outcomes *	Physical Outcomes **	QoL/Psychological Measures	Readmissions	LOS	Mortality
HOMEFOOD [48,49,56,58]; Iceland	RCT	106	Multicomponent (food + nutrition therapy and monitoring by clinical nutritionist) 24 weeks	+	+, (+), O	+, (+)	+	+	+	O
Buys et al., 2017; USA [57]	RCT	24	Food-based 10 days	+	N/A	N/A	N/A	O	N/A	N/A
Compher et al., 2025; USA [63]	Crossover RCT	46	Food-based 4 weeks	N/A	+, O	N/A	N/A	O	N/A	N/A
Howick et al., 2019; USA [62]	Pilot	10	Food-based 4 weeks	(+)	O	N/A	N/A	(+)	N/A	N/A
Lindhardt and Nielsen., 2017; Denmark [61]	Mixed methods	25	Multicomponent (food + technology support and monitoring) 12 weeks	N/A	(+)	(+)	(+)	(+)	N/A	(+)
Part of ELDORADO project—Munk et al., 2021; Denmark [47]	RCT	191	Multicomponent (dietetic care + ONS + food package) Food package 1 day only *** (Full program 16-weeks)	+	+	+	+	(-)	+ (re-admission LOS)	O
Part of ELDORADO project—Svendsen et al., 2021; Denmark [59]	Sensory evaluation	25	Food-based	N/A	N/A	N/A	N/A	N/A	N/A	N/A
Theilla et al., 2022; Israel [60]	RCT	60	Multicomponent (food + social support) 6 months	N/A	N/A	(+)	+, O	O	N/A	+

+ = significant improvement for outcome/all outcomes in category, (+) = non-significant improvement for outcome/all outcomes in category, O = no apparent effect for outcome/all outcomes in category, (-) = non-significant poor effect for outcome/all outcomes in category. More than 1 symbol means not all outcomes in that category had the same impact. N/A = not applicable as outcome not measured in that study. * Including anthropometric outcomes (body weight, BMI, body composition, calf circumference, midarm circumference), nutritional status, ADL status associated with greater weight loss and lower nutritional risk, sarcopenia scores, nutrition risk, nutrition screening. ** Including physical function, hand grip strength, 30s chair stand, functional status. *** Note this study only has a very small food-based component; the outcomes and results are more likely to be influenced by other components of the intervention.

## Data Availability

No new data were created or analysed in this study. Data sharing is not applicable to this article.

## Supplementary Materials

The following supporting information can be downloaded at: https://www.mdpi.com/article/10.3390/nu18172846/s1, File S1: Search strategies; File S2: Summary of included studies.

## Abbreviations

The following abbreviations are used in this manuscript:

BMI	Body Mass Index
ESPEN	European Society for Clinical Nutrition and Metabolism
ONS	Oral Nutritional Supplements
RCT	Randomised controlled trial
VAS	Visual Analogue Scale
