# Supplementary material for: Food-Based Nutrition Care Provided to Older Community-Dwelling Adults with or at Risk of Malnutrition Following Hospital Discharge—A Scoping Review
Source: Nutrients. 2026 Aug 31;18(17):2846. doi: 10.3390/nu18172846 (PMC13567322; doi:10.3390/nu18172846)
Supplement: Supplementary file 1 [file nutrients-18-02846-s001.zip › nutrients-4438253-supplementary.pdf]

File S1. Search Strategies

Table S1. Ovid MEDLINE (R).

No.	Query
1	Malnutrition/ or Severe Acute Malnutrition/ or Protein-Energy Malnutrition/ or Nutritional Status/ or Cachexia/ or Sarcopenia/
2	(malnourish* or malnutrition* or mal nourish* or mal nutrition* or undernutrition* or undernourish* or under nutrition* or under nourish* or cachexia or cachectic or sarcopeni* or (nutrition* adj3 (risk* or status or declin*))).ti,ab,kf.
3	1 or 2
4	Patient Discharge/ or “Continuity of Patient Care”/ or Aftercare/ or “Hospital to Home Transition”/ or Transitional Care/
5	(discharg* or postdischarg* or post hospital or posthospital or aftercare or after care or followup care or follow up care or (continu* adj3 care) or hospital to* home or (transition* adj2 care)).ti,ab,kf.
6	4 or 5
7	exp Food/ or exp Diet/ or Nutrition Therapy/ or exp Diet Therapy/ or Nutritional Support/ or Cooking/ or Eating/ or Feeding Behavior/
8	(food* or feed* or fed or eating or eat or eats or eaten or ate or meal* or diet or diets or breakfast* or lunch* or dinner* or shop* or cook* or ((diet* or nutri* or malnourish* or malnutrition* or mal nourish* or mal nutrition* or undernutrition* or undernourish* or under nutrition* or under nourish* or cachexia or cachectic or sarcopeni*) adj3 (intervent* or treatment* or therap* or support* or care or management or plan*))).ti,ab,kf.
9	7 or 8
10	3 and 6 and 9

Table S2. CINAHL Complete (EBSCOhost).

No.	Query
S1	(MH "Malnutrition+") OR (MH "Protein-Energy Malnutrition+") OR (MH "Nutritional Status") OR (MH "Cachexia") OR (MH "Sarcopenia")
S2	TI (malnourish* OR malnutrition* OR "mal nourish*" OR "mal nutrition*" OR undernutrition* OR undernourish* OR "under nutrition*" OR "under nourish*" OR cachexia OR cachectic OR sarcopeni* OR (nutrition* N2 (risk* OR status OR declin*))) OR AB (malnourish* OR malnutrition* OR "mal nourish*" OR "mal nutrition*" OR undernutrition* OR undernourish* OR "under nutrition*" OR "under nourish*" OR cachexia OR cachectic OR sarcopeni* OR (nutrition* N2 (risk* OR status OR declin*)))
S3	S1 OR S2
S4	(MH "Patient Discharge+") OR (MH "Continuity of Patient Care+") OR (MH "After Care") OR (MH "Hospital to Home Transition") OR (MH "Transitional Care")
S5	TI (discharg* OR postdischarg* OR "post hospital" OR posthospital OR aftercare OR "after care" OR "followup care" OR "follow up care" OR (continu* N2 care) OR "hospital to* home" OR (transition* N1 care)) OR AB (discharg* OR postdischarg* OR "post hospital" OR posthospital OR aftercare OR "after care" OR "followup care" OR "follow up care" OR (continu* N2 care) OR "hospital to* home" OR (transition* N1 care))
S6	S4 OR S5
S7	(MH "Food+") OR (MH "Diet+") OR (MH "Diet Therapy+") OR (MH "Nutritional Support") OR (MH "Home Nutritional Support") OR (MH "Cooking+") OR (MH "Eating") OR (MH "Eating Behavior+")
S8	TI (food* OR feed* OR fed OR eating OR eat OR eats OR eaten OR ate OR meal* OR diet OR diets OR breakfast* OR lunch* OR dinner* OR shop* OR cook* OR ((diet* OR nutri* OR malnourish* OR malnutrition* OR "mal nourish*" OR "mal nutrition*" OR undernutrition* OR undernourish* OR "under nutrition*" OR "under nourish*" OR cachexia OR cachectic OR sarcopeni*) N2 (intervent* OR treatment* OR therap* OR support* OR care OR management OR plan*))) OR AB (food* OR feed* OR fed OR eating OR eat OR eats OR eaten OR ate OR meal* OR diet OR diets OR breakfast* OR lunch* OR dinner* OR shop* OR cook* OR ((diet* OR nutri* OR malnourish* OR malnutrition* OR "mal nourish*" OR "mal nutrition*" OR undernutrition* OR undernourish* OR "under nutrition*" OR "under nourish*" OR cachexia OR cachectic OR sarcopeni*) N2 (intervent* OR treatment* OR therap* OR support* OR care OR management OR plan*)))
S9	S7 OR S8
S10	S3 AND S6 AND S9

Table S3. APA PsycInfo (EBSCOhost)

No.	Query
S1	DE “Nutritional Deficiencies” OR DE “Protein Deficiency Disorders” OR DE “Cachexia”
S2	TI (malnourish* OR malnutrition* OR “mal nourish*” OR “mal nutrition*” OR undernutrition* OR undernourish* OR “under nutrition*” OR “under nourish*” OR cachexia OR cachectic OR sarcopeni* OR (nutrition* N2 (risk* OR status OR declin*))) OR AB (malnourish* OR malnutrition* OR “mal nourish*” OR “mal nutrition*” OR undernutrition* OR undernourish* OR “under nutrition*” OR “under nourish*” OR cachexia OR cachectic OR sarcopeni* OR (nutrition* N2 (risk* OR status OR declin*))) OR KW (malnourish* OR malnutrition* OR “mal nourish*” OR “mal nutrition*” OR undernutrition* OR undernourish* OR “under nutrition*” OR “under nourish*” OR cachexia OR cachectic OR sarcopeni* OR (nutrition* N2 (risk* OR status OR declin*)))
S3	S1 OR S2
S4	DE “Hospital Discharge” OR DE “Psychiatric Hospital Discharge” OR DE “Aftercare” OR DE “Discharge Planning” OR DE “Continuum of Care”
S5	TI (discharg* OR postdischarg* OR “post hospital” OR posthospital OR aftercare OR “after care” OR “followup care” OR “follow up care” OR (continu* N2 care) OR “hospital to* home” OR (transition* N1 care)) OR AB (discharg* OR postdischarg* OR “post hospital” OR posthospital OR aftercare OR “after care” OR “followup care” OR “follow up care” OR (continu* N2 care) OR “hospital to* home” OR (transition* N1 care)) OR KW (discharg* OR postdischarg* OR “post hospital” OR posthospital OR aftercare OR “after care” OR “followup care” OR “follow up care” OR (continu* N2 care) OR “hospital to* home” OR (transition* N1 care))
S6	S4 OR S5
S7	DE “Eating Attitudes” OR DE “Food Intake” OR DE “Weight Control” OR DE “Food” OR DE “Food Preferences” OR DE “Healthy Eating” OR DE “Vegetarian Diet” OR DE “Eating Behavior” OR DE “Vegan Diet” OR DE “Meat Consumption” OR DE “Dietary Treatment” OR DE “Diets” OR DE “Food Preparation” OR DE “Nutrition”
S8	TI (food* OR feed* OR fed OR eating OR eat OR eats OR eaten OR ate OR meal* OR diet OR diets OR breakfast* OR lunch* OR dinner* OR shop* OR cook* OR ((diet* OR nutri* OR malnourish* OR malnutrition* OR “mal nourish*” OR “mal nutrition*” OR undernutrition* OR undernourish* OR “under nutrition*” OR “under nourish*” OR cachexia OR cachectic OR sarcopeni*) N2 (intervent* OR treatment* OR therap* OR support* OR care OR management OR plan*))) OR AB (food* OR feed* OR fed OR eating OR eat OR eats OR eaten OR ate OR meal* OR diet OR diets OR breakfast* OR lunch* OR dinner* OR shop* OR cook* OR ((diet* OR nutri* OR malnourish* OR malnutrition* OR “mal nourish*” OR “mal nutrition*” OR undernutrition* OR undernourish* OR “under nutrition*” OR “under nourish*” OR cachexia OR cachectic OR sarcopeni*) N2 (intervent* OR treatment* OR therap* OR support* OR care OR management OR plan*))) OR KW (food* OR feed* OR fed OR eating OR eat OR eats OR eaten OR ate OR meal* OR diet OR diets OR breakfast* OR lunch* OR dinner* OR shop* OR cook* OR ((diet* OR nutri* OR malnourish* OR malnutrition* OR “mal nourish*” OR “mal nutrition*” OR undernutrition* OR undernourish* OR “under nutrition*” OR “under nourish*” OR cachexia OR cachectic OR sarcopeni*) N2 (intervent* OR treatment* OR therap* OR support* OR care OR management OR plan*)))

S9	S7 OR S8
S10	S3 AND S6 AND S9

Table S4. Embase (Elsevier)

No.	Query
#1	'malnutrition'/de OR 'cachexia'/de OR 'sarcopenia'/de OR 'protein deficiency'/exp OR 'nutritional status'/de
#2	malnourish*:ti,ab,kw OR malnutrition*:ti,ab,kw OR 'mal nourish*':ti,ab,kw OR 'mal nutrition*':ti,ab,kw OR undernutrition*:ti,ab,kw OR undernourish*:ti,ab,kw OR 'under nutrition*':ti,ab,kw OR 'under nourish*':ti,ab,kw OR cachexia:ti,ab,kw OR cachectic:ti,ab,kw OR sarcopeni*:ti,ab,kw OR ((nutrition* NEAR/3 (risk* OR status OR declin*)):ti,ab,kw)
#3	#1 OR #2
#4	'hospital discharge'/exp OR 'aftercare'/mj/exp OR 'hospital to home transition'/de OR 'transitional care'/de
#5	discharg*:ti,ab,kw OR postdischarg*:ti,ab,kw OR 'post hospital':ti,ab,kw OR posthospital:ti,ab,kw OR aftercare:ti,ab,kw OR 'after care':ti,ab,kw OR 'followup care':ti,ab,kw OR 'follow up care':ti,ab,kw OR ((continu* NEAR/3 care):ti,ab,kw) OR 'hospital to* home':ti,ab,kw OR ((transition* NEAR/2 care):ti,ab,kw)
#6	#4 OR #5
#7	'food'/exp OR 'diet'/exp OR 'diet therapy'/exp OR 'cooking'/exp OR 'food intake'/de OR 'eating'/de OR 'fish consumption'/de OR 'fruit consumption'/de OR 'meal size'/de OR 'meat consumption'/de OR 'portion size'/de OR 'vegetable consumption'/de OR 'feeding behavior'/exp
#8	food*:ti,ab,kw OR feed*:ti,ab,kw OR fed:ti,ab,kw OR eating:ti,ab,kw OR eat:ti,ab,kw OR eats:ti,ab,kw OR eaten:ti,ab,kw OR ate:ti,ab,kw OR meal*:ti,ab,kw OR diet:ti,ab,kw OR diets:ti,ab,kw OR breakfast*:ti,ab,kw OR lunch*:ti,ab,kw OR dinner*:ti,ab,kw OR shop*:ti,ab,kw OR cook*:ti,ab,kw OR (((diet* OR nutri* OR malnourish* OR malnutrition* OR 'mal nourish*' OR 'mal nutrition*' OR undernutrition* OR undernourish* OR 'under nutrition*' OR 'under nourish*' OR cachexia OR cachectic OR sarcopeni*) NEAR/3 (intervent* OR treatment* OR therap* OR support* OR care OR management OR plan*)):ti,ab,kw)
#9	#7 OR #8
#10	#3 AND #6 AND #9
#11	#3 AND #6 AND #9 AND ([article]/lim OR [article in press]/lim OR [review]/lim)

Table S5. CENTRAL (Wiley)

No.	Query
#1	[mh ^Malnutrition] OR [mh ^"Severe Acute Malnutrition"] OR [mh ^"Protein-Energy Malnutrition"] OR [mh ^"Nutritional Status"] OR [mh ^Cachexia] OR [mh ^Sarcopenia]
#2	(malnourish* OR malnutrition* OR (mal NEXT nourish*) OR (mal NEXT nutrition*) OR undernutrition* OR undernourish* OR (under NEXT nutrition*) OR (under NEXT nourish*) OR cachexia OR cachectic OR sarcopeni* OR (nutrition* NEAR/3 (risk* OR status OR declin*))):ti,ab,kw
#3	#1 OR #2
#4	[mh ^"Patient Discharge"] OR [mh ^"Continuity of Patient Care"] OR [mh ^Aftercare] OR [mh ^"Hospital to Home Transition"] OR [mh ^"Transitional Care"]
#5	(discharg* OR postdischarg* OR "post hospital" OR posthospital OR aftercare OR "after care" OR "followup care" OR "follow up care" OR (continu* NEAR/3 care) OR "hospital to home" OR (transition* NEAR/2 care)):ti,ab,kw
#6	#4 OR #5
#7	[mh Food] OR [mh Diet] OR [mh ^"Nutrition Therapy"] OR [mh "Diet Therapy"] OR [mh ^"Nutritional Support"] OR [mh ^Cooking] OR [mh ^Eating] OR [mh ^"Feeding Behavior"]
#8	(food* OR feed* OR fed OR eating OR eat OR eats OR eaten OR ate OR meal* OR diet OR diets OR breakfast* OR lunch* OR dinner* OR shop* OR cook* OR ((diet* OR nutri* OR malnourish* OR malnutrition* OR (mal NEXT nourish*) OR (mal NEXT nutrition*) OR undernutrition* OR undernourish* OR (under NEXT nutrition*) OR (under NEXT nourish*) OR cachexia OR cachectic OR sarcopeni*) NEAR/3 (intervent* OR treatment* OR therap* OR support* OR care OR management OR plan*))):ti,ab,kw
#9	#7 OR #8
#10	#3 AND #6 AND #9 in Trials

Table S6. Search terms used for grey literature search via Google Advanced

**Search terms**
discharge malnutrition meal OR food OR "nutritional care" OR "nutritional support" site:.gov
discharge malnutrition meal OR food OR "nutritional care" OR "nutritional support" site:.gov.uk
discharge malnutrition meal OR food OR "nutritional care" OR "nutritional support" site:.gov.au
discharge malnutrition meal OR food OR "nutritional care" OR "nutritional support" filetype:pdf
discharge malnutrition meal OR food OR "nutritional care" OR "nutritional support" filetype:doc
discharge malnutrition meal OR food OR "nutritional care" OR "nutritional support" filetype:docx
discharge malnutrition meal OR food OR "nutritional care" OR "nutritional support" site:.org
discharge malnutrition meal OR food OR "nutritional care" OR "nutritional support" site:.org.uk
discharge malnutrition meal OR food OR "nutritional care" OR "nutritional support" site:.org.au

File S2

Table S7. Summary of included studies.

**Author, Year, Country**	**Design**	**Sample size**	**Intervention (and comparator)**	**Intervention duration (and follow-up)**	**Primary outcomes**	**Principal findings**
**HOMEFOOD Study (4 manuscripts) [48,49,57,59]; Iceland**.	6-month Assessor blinded RCT	n=106 (53 intervention, 53 control). Community dwelling adults ≥65 years at risk of malnutrition discharged home from hospital	**Multicomponent intervention:** Free supplemental energy- and protein-rich foods (one hot meal/day and two in-between-meals/day) + nutrition therapy by a clinical nutritionist (home visits + telehealth)<br>**Control:** Standard care including written information + recommended Meals on Wheels	Foods delivered once a week for 24 weeks<br>18 month follow up	Blondal et al. 2023a [57]<br><br>Anthropometry (body weight, BMI, body composition, calf circumference, midarm circumference), physical function, muscle strength, nutritional status, dietary intake, food-related adverse events.<br><br>Blondal et al. 2022 [49]<br>Health related quality of life, self-rated health, cognitive function, depressive symptoms, body weight, dietary intake	Blondal et al. 2023a [57]<br><br>**Intervention significantly improved** (<0.05): Anthropometric outcomes, nutritional status, physical function, dietary intake, and food satisfaction<br>**Non-significant improvements:** Handgrip strength<br>**No apparent effect:** Body fat percentage, no-food related adverse events<br><br>Blondal et al. 2022 [49]<br>**Intervention significantly improved** (<0.05): Health related QoL, self-rated health, cognitive function, depressive symptoms, body weight, dietary intake<br>**Non-significant improvements:** -<br>**No apparent effect:** -

					Blondal et al. 2023b [48]<br>Hospital readmissions (number of read-missions and length of stay, number of emergency room visits), mortality, need for long-term care residence.	Blondal et al. 2023b [48]<br>**Intervention significantly improved** (<0.05): Length of stay (6, 12, 18 months); Hospital readmissions (6, 12 months)<br>**Non-significant improvements:** Emergency room visits; risk of long-term care placement<br>**No apparent effect:** Mortality
					Eymundsdottir et al., 2025 [59]<br>ADLs (including feeding, continence, transferring, toileting, dressing, bathing, and walking), anthropometry, physical function, nutritional risk	Eymundsdottir et al., 2025 [59]<br>**Significant improvement (<0.05%):** Lower ADL status associated with greater weight loss and reduced physical function post-discharge<br>**Non-significant improvement:** Positive ADL status may be associated with lower nutritional risk<br>**No apparent effect: -**

Buys et al., 2017; USA [58]	Two-arm RCT (blinding not reported)	n=24 (12 per arm) Community dwelling adults ≥65 years at risk of malnutrition discharged to a setting where they or family/friends were responsible for meal preparation	**Food-based intervention**: usual care + education + 3 meals/day (fresh lunch, shelf-stable/refrigerated breakfast and dinner). **Control:** usual care + education only	Duration: Food delivered daily for 10 days. Follow-up: 45 days post-discharge	Hospital readmission, caloric intake, feasibility and acceptability.	**Intervention significantly improved** (<0.05): Caloric intake **Non-significant findings:** High feasibility: (87.5% retention rate) and acceptability (>80% satisfaction). Qualitative feedback highlighted meal quality, flexibility of food options; positive impact on family members; desire for more fruit and vegetables. **No apparent effect:** Hospital readmissions
Compher et al., 2025; USA [65]	Random order crossover trial	n=46 adults recently discharged from hospital	**Food-based intervention:** Seven Medically Tailored Meals (MTM) group: seven MTM (dinner only) delivered to participants home each week 21 MTM group: 21 MTM (7x breakfast, lunch and dinner) delivered to participants home each week Control: No usual-care control. Comparator alternative MTM dose.	Duration: each week for four weeks Follow-up: Baseline, day 30 and 60 following discharge from hospital.	Malnutrition risk, weight change, sarcopenia, hospital readmissions, patient experience survey	**Intervention significantly improved** (<0.05): Sarcopaenia scores **Non-significant findings:** reduced meal preparation stress, positive dietary behaviour change **No apparent effect:** Malnutrition risk, weight change, hospital readmissions

Howick et al., 2019; USA [64]	Pilot program	n=10 adults discharged home from hospital	**Food-based intervention:** One-weeks worth of meals prepared in the hospital kitchen, delivered to patients house weekly. **Control:** No comparator.	Duration: 4 wks. Follow-up: 30 days.	Nutrition screening, malnutrition risk, malnutrition prevalence, readmissions, patient reported meal consumption, patient satisfaction, and physical progress	**Intervention significantly improved**(<0.05): - **Non-significant findings:** High meal consumption (89% of each meal consumed) Patient reported improved endurance, stamina and strength to perform activities. 30-day readmission rate = 10% (compared to overall average hospital 30-day readmission rate before pilot program that was 16.3%) **No apparent effect:** Nutrition screening, prevalence of malnutrition risk, assessment and diagnoses of malnutrition not reported.
Lindhardt, and Nielsen., 2017; Denmark [63]	Mixed methods: quasi-experimental, non-randomised, controlled feasibility trial with embedded qualitative interviews	n=25 adults discharged from hospital	**Multicomponent intervention:** Protein and energy enriched meals delivered 3 x week + Tablet app used to order meals, record intake, monitor progress towards intake goals. **Control:** usual care.	Duration: 12 wks. Follow-up: Baseline, 6-weeks and 12-weeks.	Weight changes, BMI, muscle strength, health related quality of life (HRQoL), depression, readmissions, mortality, feasibility, qualitative interviews.	**Intervention significantly improved**(<0.05): - **Non-significant findings:** Improved handgrip strength, HRQoL, weight regain. Lower readmissions, deaths. Participants/relatives reported improved intake, perceived energy, reduced burden of shopping/cooking, reduced caregiver worry/conflict. Feasibility demonstrated low recruitment rates and low retention (50% intervention)

						with key barriers: exhaustion; reluctance to consume different meals from partner; incompatibility with daily routines; food preferences, satisfaction with existing meal arrangements.<br>**No apparent effect:** -
Munk et al., 2021; Denmark [60]<br><br>Svendsen et al., 2021; Denmark [61]	16 weeks single-blinded two-armed parallel randomized controlled trial<br><br>Sensory evaluation (consumer acceptance) study.	n=191 (93 intervention, 98 control) nutritionally at risk adults >50 years discharged to own home. Part of the ELDORADO project.	**Multicomponent intervention:** Individualized nutritional plan and dietary counselling + ONS script if relevant + Phone call day 4 and 30 post-discharge by hospital-based research dietitian + additional individual dietary counselling, + Week 16 post-discharge home visit + data collection + hospital discharge patients received food package to cover nutrition for first 24hrs after discharge.	Duration: Food-based intervention duration: 1 day only<br>Full program: 16 weeks.<br>Follow-up: 30 days, 16 weeks and 6 months post discharge	Readmission, length of stay of readmission, Health related quality of life, physical function, nutritional intake, use/prescription of ONS, contact of primary care provider in community, nutritional status, risk of weight loss, all-cause mortality.	Note: this study only has a very small food-based component; the following outcomes more likely to be influenced by other factors.<br>**Intervention significantly improved** (<0.05)**:** reduced length of stay for readmissions; HRQoL; physical function; nutritional intake; ONS use/prescription; contact with community services; prevention of weight loss<br>**Non-significant findings:**<br>Rates of readmission by 6 months; higher readmission rates in the intervention group at earlier follow-up points<br>**No apparent effect:** Mortality

		25 older adults receiving meals in the home setting as subset of ELDORADO project (same project as Munk et al. above)	**Control:** Standard care at discharge.<br><br>**Food-based intervention:** Food package provided to patient on ward on day of discharge. Culinary optimized meals (two main meals, two appetizers and/or desserts), (excluding supplement "goodie bag")<br>**Control:** none reported (sensory evaluation sub-study)	Duration: Not applicable<br>Follow-up: Not applicable	Sensory outcomes: Appearance, flavour, texture, appetite	**Intervention significantly improved** (<0.05)**:** -<br>**Non-significant findings:** Meals generally well accepted with neutral to positive ratings for appearance, flavour, texture, and appetite<br>**No apparent effect:** nutritional, clinical, healthcare outcomes not assessed
Theilla et al., 2022; Israel [62]	RCT	n=60 malnourished, socioeconomically disadvantaged older adults discharged home from hospital	**Multicomponent intervention:** daily dinner tray delivered to home + volunteer social support during lunch visits.<br>**Control:** Usual food intake at home	Duration: 6 mths<br>Follow-up: 3 and 6 months postdischarge	6-month survival, patient quality of life, hospital anxiety and depression scale, functional and independence status, use of resources (medication, doctor visits, hospital readmissions).	**Intervention significantly improved** (<0.05)**:** Six-month survival (87% vs 65%) Hospital Anxiety and Depression Scale (HADS) score and HADS anxiety score (3 months only)<br>**Non-significant findings:** Functional Independence Measure: positive trends at 6 months<br>**No apparent effect:** Quality of life; number of hospitalisations, emergency department visits, or

						medication changes between groups.
